# Does Post-task Declarative Learning Have an Influence on Early Motor Memory Consolidation Over Day? An fMRI Study

**DOI:** 10.3389/fnins.2018.00280

**Published:** 2018-04-26

**Authors:** Inken Rothkirch, Stephan Wolff, Nils G. Margraf, Anya Pedersen, Karsten Witt

**Affiliations:** ^1^Department of Neurology, Kiel University, University Hospital Schleswig-Holstein, Kiel, Germany; ^2^Department of Radiology, Kiel University, University Hospital Schleswig-Holstein, Kiel, Germany; ^3^Department of Psychology, Kiel University, Kiel, Germany; ^4^European Medical School Oldenburg-Groningen, Carl von Ossietzky University Oldenburg, Oldenburg, Germany

**Keywords:** SRTT, consolidation, memory system interaction, procedural learning, sequence learning, cerebellum, supplementary motor area

## Abstract

Previous studies demonstrated the influence of the post-learning period on procedural motor memory consolidation. In an early period after the acquisition, motor skills are vulnerable to modifications during wakefulness. Indeed, specific interventions such as world-list learning within this early phase of motor memory consolidation seem to enhance motor performance as an indicator for successful consolidation. This finding highlights the idea that manipulations of procedural and declarative memory systems during the early phase of memory consolidation over wakefulness may influence off-line consolidation. Using functional magnetic resonance imaging (fMRI) during initial motor sequence learning and motor sequence recall, we indirectly assess the influence of a secondary task taken place in the early phase of memory consolidation. All participants were scanned using fMRI during the learning phase of a serial reaction time task (SRTT) at 8 a.m. Afterwards, they were randomly assigned to one of five conditions. One group performed a declarative verbal, one a declarative nonverbal learning task. Two groups worked on attention tasks. A control group passed a resting condition. Participants stayed awake the whole day and performed the SRTT in the MRI scanner 12 h later at 8 p.m. At the behavioral level, the analysis of the reaction times failed to show a significant group difference. The primary analysis assessing fMRI data based on the contrast (sequence – random) between learning and retrieval also did not show any significant group differences. Therefore, our main analysis do not support the hypothesis that a secondary task influences the retrieval of the SRTT. In a more liberal fMRI analysis, we compared only the sequence blocks of the SRTT from learning to recall. BOLD signal decreased in the ipsilateral cerebellum and the supplementary motor area solely in the verbal learning group. Although our primary analysis failed to show significant changes between our groups, results of the secondary analysis could be an indication for a beneficial effect of the verbal declarative task in the early post-learning phase. A nonverbal learning task did not affect the activation within the motor network. Further studies are needed to replicate this finding and to assess the usefulness of this manipulation.

## Introduction

In 1980, Cohen and Squire postulated a classification of memory systems, describing a declarative (learning of facts) and a procedural memory system, including the learning of motor and perceptual contents (Cohen and Squire, 1980). A further division is made between conscious and unconscious learning, what is referred to as explicit and implicit memory acquisition. Both memory systems seem to interact during learning and consolidation (Poldrack and Packard, 2003). Robertson and colleagues (Robertson et al., 2004a) described memory consolidation as a phenomenon consisting of two aspects. First, the enhancement of memory performance between practice sessions, the so called “off-line” consolidation. Second, the stabilization of memory contents, which means that vulnerability to interference from a second memory task decreases with time after learning. Procedural memory contents usually consist of both implicit and explicit components, with different weighting depending on the specific structure of it. Therefore, learning a motor task is never exclusively implicit as certain aspects of it have an explicit character. The procedural as well as the declarative aspects of a previously learned content can usually be consolidated distinctly from each other only while asleep because the declarative and procedural memory systems interact during wakefulness but decouples during sleep (Walker et al., 2002; Robertson et al., 2004b; Stickgold, 2005). This interaction during wakefulness was shown by Brown and Robertson: A declarative task can interfere with the process of consolidation of the procedural memory when consolidation takes place over a period of wakefulness but not during sleep (Brown and Robertson, 2007a). This work provided evidence that learning a word list immediately after procedural motor learning in the morning leads to an improvement of motor performance in the evening. This effect is lost when consolidation takes place over night. Considering the idea of interacting memory systems, Robertson concluded that the declarative component of a motor task hinders motor consolidation: Motor memory consolidation during wakefulness is supported when the declarative part of the learning task is removed. Robertson argued that a declarative learning task performed immediately after motor learning may decouple the declarative aspects of the task (Robertson, 2009) and Brown and Robertson (2007a) demonstrated the effect of a declarative task in the early phase of motor memory consolidation. We intend (i) to replicate this finding on a behavioral level and (ii) want to indirectly investigate the changes associated with the influence of a secondary task on the motor system using fMRI. Learning and consolidation of motor sequences in healthy humans mainly comprise the cerebellum and the basal ganglia, the supplementary motor area (SMA) and premotor as well as the primary motor cortex (Hikosaka et al., 1999; Grafton et al., 2002; Ungerleider, 2002). (iii) We extend our study to assess the influence of a nonverbal learning task placed in the early phase of motor memory consolidation. Each active group (verbal learning group and nonverbal learning group) will be separately compared to control groups dealing with verbal material or nonverbal material without a significant memory component.

## Materials and methods

### Subjects

**Figure 1:**
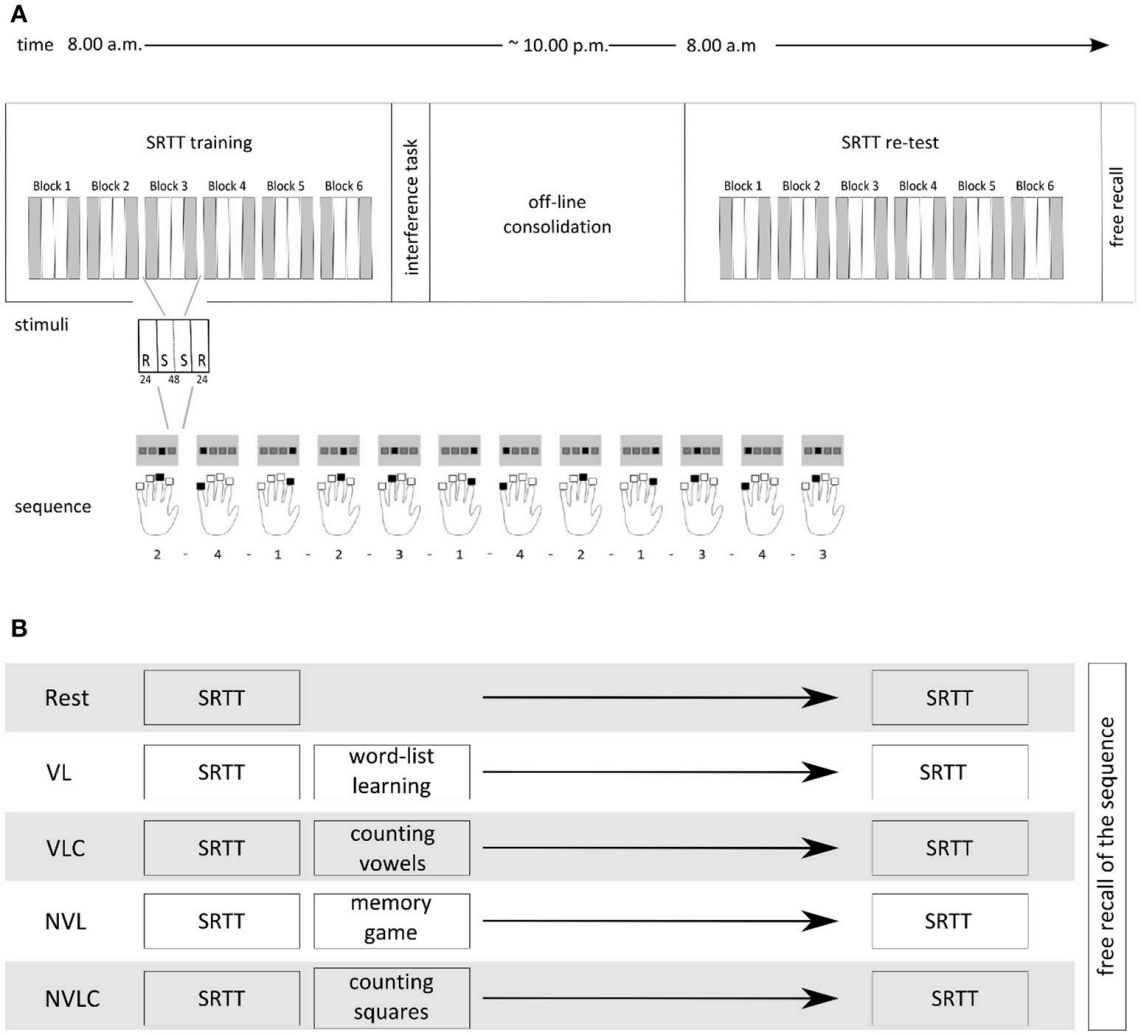
Experimental Design. **(A)** The upper part shows the experimental design including a SRTT learning session in the morning (8.00 a.m.) followed by a group specific interference task. The SRTT retest was performed in the evening (8.00 p.m.) followed by the test of explicit knowledge. Each block of the SRTT included 24 randomized trials, followed by 48 sequential trials and again 24 randomized trials. Participants responded with the fingers of the left hand to the visually presented stimuli. **(B)** Overview of the groups' naming and the appropriate inference tasks after the initial conduction of the SRTT.

Seventy-five healthy young participants (40 women) volunteered for the study. All participants were right-handed, assessed using the Edinburgh Handedness Inventory (Oldfield, 1971), had no history of neurological or psychiatric illness and gave written informed consent to take part in the study. The study was approved by the ethics committee of the University Medical Faculty in Kiel and was conducted in full accordance with the Declaration of Helsinki. All participants received an honorarium of 40 €. Participants were randomly assigned (using the sample function in R statistics) to one of five experimental groups right after their 8 a.m. scan (see below and Figure 1B), each with a sample size of *n* = 15. Due to different reasons, we excluded nine subjects from further data analyses. For three participants, problems in preprocessing occurred because of suboptimal image quality due to movement artifacts. These participants moved their heads to such an extent that brain areas relevant for imaging analysis were cut off. For one subject, the recording of the behavioral data failed. Another subject had an incidental intracerebral finding. The reaction times in the SRTT of three participants lay outside of the 95% confidence interval computed over the reaction times for correctly answered trials of all subjects and both sessions. One subject became aware of the hidden sequence. Finally, we included 66 subjects with a mean age of 24.0 ± SD 2.0 years (range: 19–29) forming five groups. The verbal learning group (VL) consists of *n* = 12 (6 women) with a mean age of 24.4 (± 1.89 SD) years, the verbal learning control (VLC) group of *n* = 14 participants (10 women) with a mean age of 24.5 (± 2.90 SD) years. For the nonverbal learning group (NVL) we could include *n* = 13 subjects (6 women) with a mean age of 23.0 (± 2.98 SD) years and for the nonverbal learning control group (NVLC) *n* = 12 participants (7 women) as well with a mean age of 23.0 (± 1.93 SD) years. Finally, the Rest group comprised *n* = 15 subjects (8 women) with a mean age of 24.0 (± 2.00 SD) years. No significant differences neither in age [*F*_(4, 61)_ = 1.06, *P* < 0.384] nor in gender ($\chi_{4}^{2}$ = 2.029, *P* < 0.73) were obtained.

### The serial reaction time task (SRTT)

All participants completed a modified version of the serial reaction time task with their non-dominant left hand (Nissen and Bullemer, 1987), i.e., a sequence motor learning task, while in a MRI-scanner at 8:00 a.m. and 8:00 p.m. of the same day. Stimulus presentation and recording of participants' reaction times were carried out with an Invivo IFIS fMRI system (Invivo, Gainesville, Florida, USA) programmed in E-Prime® software (Psychology Software Tools, INC, Sharpsburg, USA). Four horizontally arranged gray squares were presented on a monitor, each square for one of the four left-handed fingers that rested on the corresponding four-button response pad. Each trial contained 12 stimuli, namely 2-4-1-2-3-1-4-2-1-3-4-3. The squares lit up blue one at a time, either in a randomized order or in a sequential order. Random sequences did not include a duplication or repetition of the same number/lit square in a row. Participants were not told about the embedded sequence, but were asked to react to the stimuli by pressing the corresponding key on their response pad as fast and accurately as possible. Unlike Brown and Robertson (2007b), we chose a block design characterized by an alternating sequential and randomized item structure with only six blocks of learning with 144 sequential stimuli items included. For practical reasons using fMRI technique we had to change the classical setting of the SRTT. For the robustness of our statistics, we therefore needed the same number of sequential and randomized trials in a block design. Figure 1 depicts the overall procedure of the SRTT paradigm.

### Experimental groups

After performing the SRTT, participants completed different secondary tasks. The verbal learning group (VL, *n* = 12) performed the German version of the California verbal learning task (Niemann et al., 2008), a word-list task, in which participants were ask to learn 16 words within five trials. This verbal declarative and explicit learning task should activate the declarative memory system. Following the findings of Brown and Robertson (2007a), this group functions as experimental group, because due to the structure of the second task, a change in motor network activation is expected here. The verbal learning control group (VLC, *n* = 14) conducted a vowel-counting task after finishing the SRTT with the instruction to count the vowels in nonsensical strings of letters with a length between three and 12 letters. Handling with the same verbal material avoiding learning efforts, this group represents the control group for a direct comparison with the VL group. We further extend our study investigating the effect of a nonverbal learning task applied immediately after learning the SRTT. Therefore, we have also included a nonverbal condition with a direct control group. The nonverbal learning group (NVL, *n* = 12) played a computer version of the “Memory” game as a nonverbal declarative and explicit learning task, an adapted version of the task described by Rasch et al. (2007). If the learning of nonverbal material influences the early phase of motor memory consolidation, we might expect changes in cerebral activation of the motor system as well. The nonverbal learning control group (NVLC, *n* = 12) counted squares within a cloud of points, again this task focuses on attention spatial processes minimizing the memory component. The control groups differ in the material of the task, which is adapted to the respective experimental group. In the rest group (Rest, *n* = 15) participants laid down in a dimly lighted room with no further task after the SRTT. They were not allowed to sleep. This group served as control group for both experimental groups. After the morning session, all participants left our laboratory, did their daily business and returned to the laboratory in the evening. Sleeping and napping was not allowed for all groups. We implemented the NVL and NVCL groups to address the question, whether nonverbal declarative learning may have a different influence on the consolidation of procedural memory over wakefulness.

### Assessment of explicit awareness

After the SRTT retest at 8 p.m. we informed all participants about the embedded sequence and they gave a verbal free recall of the sequence. Participants had to write down 12 elements guessing the implicitly learned sequence on a prepared sheet. To control for explicit knowledge of the sequence we split the recalled items into triplets. Sequence is modeled like a periodic signal. Therefore, the second to the last and the last item of the original sequence were also fit together with the first, or respectively, the first two items of the sequence. Subjects could maximally reach a score of 12 correct triplets. The binomial distribution (*n* = 12), that can be used to describe the scoring of completely randomly selected sequences (hence performance), was determined by a scoring probability of 0.33 because 36 triplets were possible when repetitions of the same number in a row were not allowed. Consequently, the critical value for significance (*p* < 0.05) is equal to a score of seven correctly recalled triplets. We chose this value as the cut-off point, with the interpretation that subjects recalling seven or more correct triplets had a significant gain of explicit sequence knowledge.

### MRI data acquisition

Anatomical and functional images were acquired in the Neurocenter at Kiel University Hospital with a 3T whole-body MRI scanner (Achieva 3T, Philips, Best, The Netherlands) provided with an 8-channel head coil. An IFIS system (Invivo, Gainesville, Florida, USA) was applied for stimulus presentation and response recording. For functional MRI a whole-brain echo planar imaging (EPI) sequence with the following parameters was used: repetition time (TR) = 2.4 ms, echo time (TE) = 36.4 ms, field of view (FOV) = 216 × 216 mm^2^, flip angle = 90°, matrix = 64 × 64, volumes = 330, slices = 36, slice thickness = 3.0 mm, and inter-slice gap = 0.3 mm. The axial slices were acquired parallel to the anterior-posterior plane. For all subjects, additional three-dimensional (3D) T1-weighted gradient echo MRI scans with sagittal volume excitation were acquired with the following parameters: TR = 8.2 ms, TE = 3.7 ms, FOV = 240 × 240 mm^2^, flip angle = 8°, slices = 160, matrix = 240 × 240, voxel size = 1 × 1 × 1 mm^3^. T2 axial scans and a FLAIR sequence were performed to screen for structural abnormalities.

### Analysis of imaging data and preprocessing

For image preprocessing and functional analysis the SPM12 (Release 6225) software package (SPM12; Wellcome Department of Imaging Neuroscience, London, http://www.fil.ion.ucl.ac.uk) as well as Matlab Version 8.5 (R2015a) (MathWorks Inc., Natick, Massachusetts, USA) were used. In a first step of the preprocessing, all functional EPI images were realigned to compensate for subjects' movements during the scanning. The anatomical T1-weighted images were then spatially normalized to the standard coordinates of the Montreal Neurological Institute (MNI) space. The realigned EPI images were then co-registered with the corresponding individual T1-weighted image. By concatenating these two steps, the SPM software wrote normalized versions of the EPI images (2 × 2 × 2 mm^3^) allowing a voxel wise analysis of the BOLD time-series and statistical group comparisons. Finally, we smoothed the functional MRI data with a Gaussian kernel filter of 8 mm full-width at half-maximum (FWHM).

### Statistical analysis

#### Behavioral data of the sequential motor task

Performance in the SRTT was measured as the response time that was needed to press the button after a stimulus was presented (Robertson, 2007). Thereby, implicit sequence learning can be defined as the difference in mean response times between sequential and the subsequent randomized stimuli. Since sequence learning progresses over time, the second phase of sequential trails (R1-S1-**S2**-R2) and its contrast to the immediately followed random trails (R1-S1-S2-**R2**) should better reflect motor learning than the mean over all sequential stimuli (S1 and S2) contrasted to all random stimuli (R1 and R2). Consequently, we operationalized off-line consolidation as the difference between R2 and S2. As performance parameters we defined skill1 = R2-S2 for the learning phase and skill2 = R2-S2 for retesting. As the overall measure for performance gain after off-line consolidation we computed delta-skill = skill2–skill1. A further methodological approach for operationalizing the effect of off-line consolidation on implicit sequence learning is the comparison of the last SRTT block at learning with the first SRTT block at retest. The definition of performance parameters remained unchanged. To investigate possible performance differences in skill1, skill2 and delta-skill between the five experimental groups, we first conducted an analysis of variance (ANOVA) with repeated measures with *group* as between-subject factor and skill consisting of skill1 and skill2 as repeated measures factor. Further, we computed a second one-way ANOVA with *group* as between-subject factor and delta-skill as dependent variable. Both analyses were conducted for skill data over all learning and retest blocks as well as for the comparison between the last block at learning and the first block at retest. We only included response times of correct responses in the statistical analysis. Wrong button presses as well as missed button presses were treated as an error. The mean error rate was 3.47 ± 1.95%. Because of this ground effect, we did not include this variable to further analysis. Statistical analysis was conducted in R statistics (R Core Team, 2016).

#### Functional analysis (fMRI)

Time-series analysis (first level) of the functional imaging data was designed to apply a general linear model (GLM) with two regressors for sequential stimuli as well as two for randomized stimuli according to the structure of our paradigm. We decided to model our data analysis with these four regressors of interest, as this allows the differentiation between R1-S1-S2-R2 (Figure 1). The six movement associated parameters resulting from the realignment process were included as regressors of no interest. The models were characterized by a boxcar function corresponding to the effects of interest and convolved with the standard canonical hemodynamic response function implemented in SMP12.

First, we computed a GLM, including only the learning session, to look into the main effect of sequential trials and check for group differences. Further, this model helped us to define those areas that are activated under sequence learning and show a significant main effect at learning session over all subjects. We refer to those regions as motor network. As our aim was mainly the investigation of a change in functional imaging initiated by different interference tasks after an initial motor task, we were interested in the group by time interaction (effect of interest) concerning brain areas belonging to the motor network. On the group level (second level analysis), we therefore analyzed both sessions (training and retesting) in a common GLM. In a first step, we computed the group level analysis on the same contrast that we used on behavioral level (delta-skill), namely (R2_T1_-S2_T1_) − (R2_T0_-S2_T0_). However, we found no effect for this contrast. This result leads to two different possible interpretations: (1) no effect is evident between sessions and (2) the BOLD signal of the randomized trials overpowered the activation of sequential stimuli. On the assumption that the second interpretation is correct, we can analyse the contrast (*S2*_*T*1_ > *baseline*_*T*1_*)* − *(S2*_*T*0_ > *baseline*_*T*0_*)*. If we got a significant group-by-time interaction based on this contrast, we can actually conclude, that the second assumption is correct because otherwise this contrast also should have led to no significant effects. Therefore we based our analysis on the first level contrast (*S2*_*T*1_ > *baseline*_*T*1_*)* − *(S2*_*T*0_ > *baseline*_*T*0_*)* whereby the baseline was the time between two blocks of training and retesting respectively. As Brown and Robertson (2007b) found evidence for differences in the consolidation process after conducting a declarative or a procedural task, our special interest focused on the comparison of the VL and the VLC group. For the analysis of the group by time interaction we searched for all motor sequence learning associated brain areas (cluster size: *k* ≥ 10) that reached significance in the contrast reflecting the *group* × *time* interaction for all voxels in the whole brain (*P* < 0.05; FWE corrected). We then extracted the time series for all significant voxels within a sphere of 4 mm around the peak location of each motor network area. Whether a significant voxel cluster belonged to a motor network area was verified using the SPM Anatomy Toolbox (Version 2.2c). Following this step, we were able to conduct Wilcoxon signed rank tests to look for differences between two groups at a time.

## Results

### Behavioral results

#### Performance analysis of the serial reaction time task over all SRTT blocks

**Figure 2:**
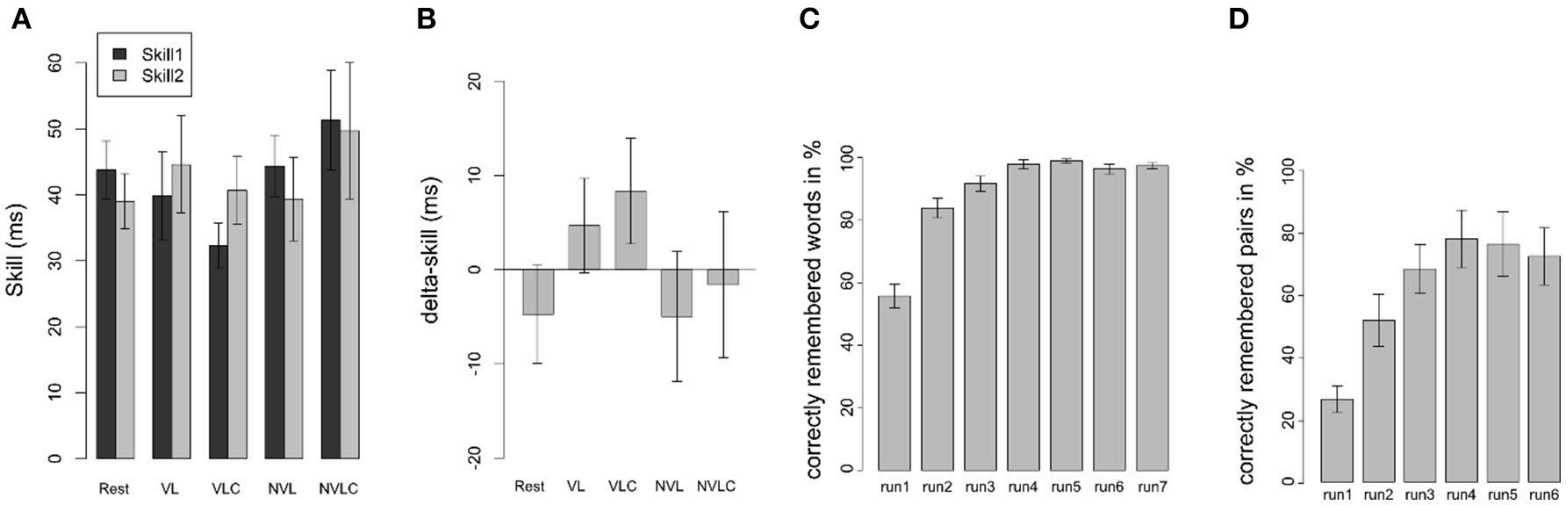
Behavioral results of the SRTT. **(A)** Behavioral data of the SRTT are shown as mean differences in reaction times (±SEM) between randomized and sequential stimuli in the learning (skill1, dark bars) and the retest sessions (skill2, light bars) corresponding to the five experimental groups (Rest, VL, VLC, NVL, NVLC). Panel **(B)** displays the performance gain after 12 h of offline consolidation (delta-skill = skill2 - skill1). Panel **(C)** gives an overview of the performance of the CVLT in the VL group. Each bar reflects the mean (±SEM) of one learning run of the word list. Panel **(D)** shows the performance of the memory game in the NVL group. Each bar reflects the mean (±SEM) of one learning run of the game. (Rest, Rest group; VL, verbal learning group; VLC, verbal learning control group; NVL, nonverbal learning group; NVLC, nonverbal learning control group).

The ANOVA with the between-subject factor *group* (VL, VCL, NVL, NVLC, Rest) and *skill* (skill1, skill2) as within-subject factor revealed neither significant main effects for skill [*F*_(1, 120)_ = 0.063; *P* = 0.802, Figure 2A] nor group [*F*_(4, 120)_ = 1.309; *P* = 0.271, Figure 2A]. In addition, no significant group-by-skill interaction was found [*F*_(4, 120)_ = 0.755; *P* = 0.557]. Therefore, group performance in the SRTT was not different between learning and retest and did not differ between groups. However, learning was evident over all participants, as response times for randomized stimuli were higher than for sequential stimuli in skill1 [*t*_(65)_ = 17.29; *P* < 0.001] as well as in skill2 [*t*_(65)_ = 14.47; *P* < 0.001]. Further, we found no differences in off-line consolidation between the groups, as the ANOVA for delta-skill did not become significant [*F*_(4, 61)_ = 0.977; *P* = 0.427, Figure 2B]. In concordance with the fMRI analysis, we computed in a second step the contrast *S2*_*T*1_ > *S2*_*T*0_ on behavioral level. Thus, we based this analysis on the second phase of sequential trails in each block (R1-S1-**S2**-R2) and compared the reaction times between learning and retest. However, this method leads to no significant main effect for *group* [*F*_(4, 61)_ = 0.415; *P* = 0.797]. The aforementioned variations between our design and the experimental structure used by Brown and Robertson (2007b) hindered the replication of the behavioral results seen by Brown and Robertson (2007b).

#### Performance analysis of the serial reaction time task over the last block at learning and the first block at retest

As a further methodological approach for operationalizing implicit sequence learning, we compared the last SRTT block at learning with the first SRTT block at retest. The ANOVA including the between-subject factor *group* (VL, NVL, VLC, NVLC, Rest) and the within-subject factor *skill* (skill1, skill2) revealed neither significant main effects for skill [*F*_(1, 120)_ = 2.697; *P* = 0.103] nor group [*F*_(4, 120)_ = 0.785; *P* = 0.537]. Again, group performance in the SRTT was not different between learning and retest and did not differ between groups. Further, we found no differences in off-line consolidation between the groups, as the ANOVA for delta-skill did not become significant [*F*_(4, 61)_ = 1.482; *P* = 0.219].

#### Performance analyses of the interference tasks

To ensure that declarative learning was successful, we analyzed the number of learned words in the VL group. An ANOVA with repeated measurements showed a significant learning gain [VL: *F*_(4, 54)_ = 38.16; *P* < 0.001, Figure 2C]. Therefore, declarative learning was successful. In the control task participants responded with the number of correctly counted vowels (VLC) at a rate of 97% (±2.08). Overall, we assume a successful performance of the interference tasks in both groups. For the NVL group the ANOVA for a learning effect in the memory game did not reveal significance due to the ceiling effect of performance [*F*_(5, 60)_ = 0.99; *P* = 0.434, Figure 2D]. The mean percentage of correctly counted squares in the NVLC group was 97% (±2.82) and therefore comparable to the performance in the VLC group.

#### Awareness of the sequential structure

As we investigated implicit learning and its consolidation, we had to make sure that subjects did not become aware of the hidden sequence within the SRTT. When subjects were asked directly after the retest execution of the SRTT if they noticed any sequential pattern within the task, all participants answered “no” to the question, with the exception of the participant who has acquired explicit knowledge. The mean score of correctly replicated triplets was 2.96 with a standard deviation of 1.34, which is within the range of chance performance. No significant group differences in the awareness of the sequential structure were found [M_Rest_ = 3.5, M_VL_ = 3.08, M_VLC_ = 2.67, M_NVL_ = 3.25, M_NVLC_ = 3.5, *F*_(4, 61)_ = 0.216; *P* = 0.928].

### Functional imaging results of the serial reaction time task

#### The impact of an interference task on motor sequence consolidation

**Figure 3:**
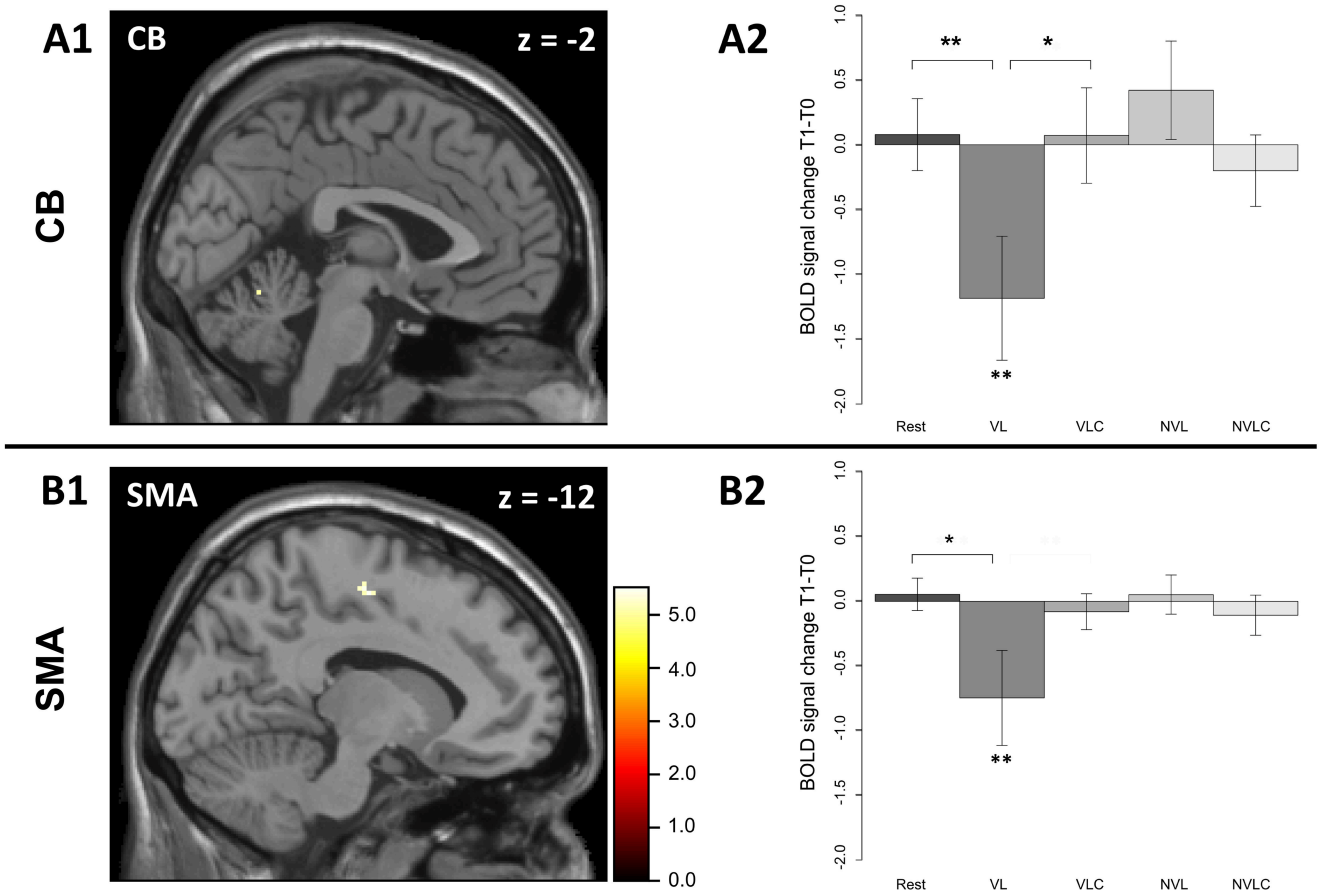
Significant regions in the group × time interaction in the sequence > baseline contrast. The group (VL and VLC) x time (learning and retest) interaction results in a significant effect in the local maximum in **(A)** the left cerebellum (**A1**, −2 −60 −14) and in the local maximum in **(B)** the left SMA (**B1**, −12 −12 52) [FWE corrected]. The VL group shows a significant change in the BOLD signal between learning and retesting (**A2**, CB; *W* = 69, *P* < 0.013; **B2**, SMA; *W* = 68, *P* < 0.021). The error bars in **A2** and **B2** display standard errors of mean. *P* < 0.05 ^**^, *P* < 0.1 ^*^; Rest, Rest group; VL, verbal learning group; VLC, verbal learning control group; NVL, non-verbal learning group; NVCL, non-verbal learning control group.

Our special interest focused on differences between the VL and the VLC group. In a first step, we computed the group level analysis on the same contrast that we used on behavioral level (delta-skill), namely (R2_T1_-S2_T1_) – (R2_T0_-S2_T0_). However, we found no effect for this contrast. Our second and more liberal analysis was based on the first level contrast (*S2*_*T*1_ > *baseline*_*T*1_*) – (S2*_*T*0_ > *baseline*_*T*0_*)* whereby the baseline was the time between two blocks of training and retesting respectively. This resulted in a significant *group* × *time* interaction within the left cerebellum (CB) (Figure 3A1) and left supplementary motor area (SMA) (Figure 3B1). A closer look at this interaction, including all groups, revealed the following: In contrast to the other groups, the VL group showed a significant change in BOLD signal in the cerebellum as well as in the supplementary motor area (SMA) comparing the learning to the retesting sessions. The activation in both areas decreased (CB: *W* = 10, *P* = 0.021; SMA: *W* = 14, *P* = 0.053; Figures 3A2,B2). Planned contrast analysis (Table 1) demonstrated a significant difference in BOLD signal changes in the cerebellum from learning to retest between the VL group and the Rest group. Further, a trend for this BOLD signal changes in the cerebellum was found between the VL and the VLC group (Figure 3A2). For the SMA we found a similar pattern (Figure 3B2) for the VL group-Rest comparison.

**Table 1:** Pairwise *post hoc* Wilcoxon-Tests for group differences in BOLD signal change between learning and retesting.

**Compared groups**	**Cerebellum [**−**2** −**6** −**14]**		**Supplementary Motor Area [**−**12** −**12 52]**	
	***W*-value**	***P*-value**	***W*-value**	***P*-value**
VL – Rest	133	0.037	127	0.075
VL – VLC	50	0.085	67	0.403
Rest – VLC	108	0.915	130	0.289
NVL – Rest	82	0.496	92	0.821
NVL – NVLC	93	0.437	90	0.538
Rest – NVLC	97	0.755	107	0.427

*Coordinates are given in MNI space*.

#### The main BOLD effect of sequential trials before consolidation

The main effect of the sequential trials (S2) showed an increased BOLD signal in the precentral gyrus bilaterally, as well as in the cerebellum, supplementary motor area (SMA), postcentral gyrus and putamen in the left hemisphere (for details see Table 2). We masked the statistical map at all areas associated with the motor system bilaterally, resulting in significant activations (*p* < 0.01, FWE corrected) in the bilateral postcentral and precentral gyri, supplementary motor area, caudate nucleus as well as in the left cerebellum (lobule 4 and 5) and putamen. Masking was done by Neuromorphometrics' Probabilistic Atlas (Neuromorphometrics, Inc., Somerville, Massachusetts, USA). We found no significant differences on the whole brain analysis (FWE corrected) in the BOLD signal when comparing the groups at the learning session. After reducing the group comparison to motor system related areas, also no significant differences were found (FWE corrected).

**Table 2:** Main effects in sequential trials in all subjects at the learning session.

	**Left Hemisphere**					**Right Hemisphere**				
**AAL-Region**	**x**	**y**	**z**	***t*-value**	**Cluster size**	**x**	**y**	**z**	***t*-value**	**Cluster size**
Precentral	−50	0	42	11.97	10186	58	6	32	6.28	10186
Postcentral	−62	14	32	5.60	2479					
Parietal_Inf	−32	−58	54	11.17	2479	30	−50	48	7.50	10186
Parietal_Sup	−26	−62	58	12.00	2479	26	−60	52	8.07	10186
Frontal_Mid	−24	−6	52	14.08	10186					
Occipital_Mid	−42	−70	4	11.47	838	30	−70	28	6.70	10186
Supp Motor Area	−4	2	58	14.00	10186					
Cerebellum_4_5_L	−12	−52	−22	9.46	612					
Caudatus	−10	18	−4	5.51	420	10	24	0	5.29	17
Putamen	−22	0	6	6.93	266					
Thalamus	−10	−14	2	5.01	335	16	−14	6	5.07	1

*Whole brain local cluster peaks after correction for multiple comparisons (P < 0.05; FWE corrected)*.

*Coordinates are given in MNI space*.

## Discussion

In this study, we investigated the changes between the learning phase and the retrieval of modified version of the SRTT (Nissen and Bullemer, 1987). Five groups differ in terms of a post-learning task following the initial SRTT. Using this study design, we only are able to assess the consolidation phase indirectly and we are not able to examine specific mechanisms that took place in the early phase of motor memory consolidation. Two main results are evident. First, we found no significant performance differences among our five groups. Second, assessing fMRI data based on the contrast (sequence − random) between learning and retrieval also do not show any significant group differences. Therefore, our main analysis does not support the hypothesis that a secondary task influences the retrieval of the SRTT.

Behavioral data in our study differs from Brown's and Robertson's (2007b) results. Various explanatory approaches are conceivable. Foremost, we specifically reprogrammed the classical SRTT to make it suitable for an fMRI setting. In the Brown and Robertson study, participants repeated the sequence in the learning phase more often (55 repetitions of the sequence) than in the present study (24 repetitions of the sequence). Furthermore, the sequential and random stimuli switched more often in our study compared to the Brown and Robertson study. These differences could have far-reaching effects on the SRTT outcome. A study by Schendan et al. used a comparable version of the SRTT with alternating sequential and randomized blocks conducted in a MRI scanner (Schendan et al., 2003). The reaction times for both randomized and sequential stimuli in their last SRTT block are comparable with the reaction times that we found in the last block of our training session in the morning. Tzvi et al. conducted the same version of the SRTT as we did, again in a MRI scanner (Tzvi et al., 2015). Behavioral performance in the learning phase was comparable over all blocks and subjects to those presented in this study. Unfortunately, relatively few studies have been conducted using a similar kind of the SRTT as we did. However, it seems that the number of repetitions has an influence on the learning outcome. This may explain different results across different studies. Another explanatory approach for the diverging behavioral results in this study compared to Brown and Robertson (2007b) are the different sample sizes of the studies. As we conducted an fMRI experiment, we were not able to collect as much data as it is possible in a behavioral study. That is why behavioral data in our case may be underrepresented, but the quality of the imaging data should not be affected. Moreover, we have to consider two important limitations concerning the evaluation of reaction times using fMRI: First, reactions on response pads designed for fMRI experiments differ from keyboards used outside the scanner in the necessary physical effort to press a key. Second, participants in the scanner lay in a supine position instead of sitting on a chair. Both aspects lead to a situation that might not be comparable to optimized test situations outside the scanner. This might have overshadowed the behavioral advantage that has previously been reported for a verbal learning group.

Our first approach to fMRI data analysis based on the contrast (sequence – random) over both time points (learning and retrieval) resulted in no significant group differences as well. This may have been caused by the limited differences between activation in sequential and random trials. In our design random and sequential phases switches rapidly making it difficult to extract sequence specific activation. In conclusion, our primary analysis failed to show an effect of a manipulation of the post learning period. However, in a more liberal fMRI analysis comparing only the sequential trials of the SRTT, we found a significant change in cerebral activation in the verbal learning group from learning to retesting. However, the appropriate control groups (VLC and Rest) showed no such differences in cerebral activation between both sessions meaning that an attention task did not significantly influence the cerebral activation from learning to retest. The BOLD signal change in the VL group appeared as a decrease from learning to retesting in the cerebellum and the SMA. This may correspond to the memory interaction on behavioral level during off-line consolidation over wakefulness shown by Brown and Robertson (2007b). Participants who learned a word list immediately after the acquisition of a procedural skill demonstrated a better performance after off-line consolidation over day (Brown and Robertson, 2007a). In the initial phase of skill acquisition, fMRI analysis revealed an increased BOLD signal for the (Sequence > baseline) contrast in those brain areas associated with the SRTT (Hardwick et al., 2013): the left primary somatosensory cortex, putamen, thalamus, bilateral primary motor cortex, dorsal premotor cortex, supplementary motor area, as well as the right cerebellum (Lobule IV-V). The cerebral activation showed a pattern that is in line with previous reports of motor learning (Grafton et al., 2002; Daselaar et al., 2003; Hardwick et al., 2013; King et al., 2016). Moreover, we found no significant differences in brain activation when comparing the groups during the learning phase. Therefore, changes in brain activation from learning to retesting between our groups cannot depend on different activations at the starting point, namely the initial learning within the scanner. Taking these two aspects together, relativizes the insufficient replication of the behavioral data as missing baseline differences in imaging at learning and the representative functional mapping of the initial SRTT conform to our hypothesis. Further, we found evidence for sequence specific learning as the response times to sequential stimuli were shorter than to randomized ones over all subjects. These results can be interpreted as strong evidence for our data's validity.

In the verbal learning group, we found a significant BOLD signal decrease from learning to retesting not only in the cerebellum and the SMA, but also in the precentral and postcentral gyri, in the inferior and superior parietal cortex, in the middle frontal and middle occipital cortex as well as in the caudate, putamen and thalamus. Given the fact that the VL group was the only group that showed significant changes between learning and retesting in the motor network, the influence of a verbal learning task is specific and sensitive for the manipulation within the phase of off-line consolidation. However, the *group x time* interaction remained significant for two regions: the left cerebellum (Lobule IV-V) as well as the left supplementary motor area. Both areas are involved in task acquisition. Sleep dependent off-line consolidation has previously shown a shift in BOLD signal activation from primary motor areas involved in the acquisition of a motor task to prefrontal areas (Fischer, 2005; Peigneux et al., 2006). Our participants performed the SRTT with the left hand, so activation changes in the ipsilateral cerebellum are plausible because the ipsilateral and the contralateral cortical regions are mainly involved in the management of fine finger movements (Imamizu et al., 2003; Doyon et al., 2009). The SMA of the dominant hemisphere is known to be active during implicit learning tasks performed with the right but also with the left hand (Grafton et al., 1995; Hazeltine et al., 1997). Lesions affecting the SMA of the dominant hemisphere are associated with a deficit in sequence learning (Halsband et al., 1993; Ackermann et al., 1996). In conclusion, the SMA of the dominant hemisphere plays a superior role in implicit sequence learning. In the present study, our second and more liberal fMRI analysis showed a reduction of SMA activity in the VL group compared to the appropriate control conditions (VLC and Rest) that may have reflected the beneficial consequences of motor memory consolidation when both the declarative and the non-declarative memory systems were uncoupled in an experimental setting. In contrast, our results show a decline in the motor network involved in motor learning. Motor speed is associated with higher BOLD signals (Albouy et al., 2012) and we found a significantly lower activation in the retest condition, specifically in the verbal learning group. These results raise the possibility that early off-line consolidation may affect those areas involved in skill acquisition. However, we found no shift in activation to prefrontal areas. In this way, the off-line consolidation induced by an interference task differs from sleep dependent off-line consolidation (Peigneux et al., 2001; Fischer, 2005). Our data, however, apparently contradicts the results of Robertson et al. (2004b), who postulated that the consolidation of implicit learned motor skills was not sleep dependent, as skill performance improved after a consolidation phase with or without sleep. Explicitly learned motor skills, however, require sleep in the consolidation phase for their improvement. Along these lines, a consolidation effect within the resting group could has been expected because the subjects learned the sequence implicitly and consolidated it over a period of wakefulness. To solve this apparent contradiction, we assume that an interference task immediately conducted after sequence learning has a stronger effect on consolidation than the remaining time of the consolidation phase. We were not able to examine participants' activities between testing in the morning and retesting in the evening exactly. Activities done within this time might have interfered with motor memory consolidation. However, we randomly allocated the subjects to the five experimental groups after conducting the SRTT in the morning. Consequently, the groups merely differed in terms of the post-task test. Distortions that occurred due to the different activities during the consolidation phase should have been distributed equally among the groups.

To conclude, we found no relevant differences in the nonverbal learning group within the motor network. This raises the idea that early memory consolidation may be specific for the type of declarative learning, but further studies are needed to replicate this finding. Based on the results here, we cannot make any inference on the impact of nonverbal learning on the consolidation process over wakefulness.

## Author contributions

The conception of the Research Project was done by IR, KW, and NM, organization by KW and the execution by IR, NM, and SW. The design of the statistical analysis was done by IR and KW, analysis was conducted by IR. IR and KW wrote the main manuscript text and prepared the figures with the help of SW. Statistical analysis was supported and reviewed by AP, KW, NM, and SW. All authors reviewed the manuscript.

### Conflict of interest statement

The authors declare that the research was conducted in the absence of any commercial or financial relationships that could be construed as a potential conflict of interest.
